# Maresin‐1 ameliorates hypertensive vascular remodeling through its receptor LGR6

**DOI:** 10.1002/mco2.491

**Published:** 2024-03-09

**Authors:** Zheng Yin, Jishou Zhang, Mengmeng Zhao, Shanshan Peng, Jing Ye, Jianfang Liu, Yao Xu, Shuwan Xu, Wei Pan, Cheng Wei, Juan‐Juan Qin, Jun Wan, Menglong Wang

**Affiliations:** ^1^ Department of Cardiology, Renmin Hospital of Wuhan University, Department of Geriatrics, Zhongnan Hospital of Wuhan University Wuhan University Wuhan China; ^2^ Cardiovascular Research Institute Wuhan University Wuhan China; ^3^ Hubei Key Laboratory of Cardiology Wuhan China; ^4^ Center for Healthy Aging Wuhan University School of Nursing Wuhan China

**Keywords:** leucine‐rich repeat‐containing G protein‐coupled receptor 6, Maresin‐1, omega‐3 fatty acids, pyroptosis, vascular remodeling

## Abstract

Hypertensive vascular remodeling is defined as the changes in vascular function and structure induced by persistent hypertension. Maresin‐1 (MaR1), one of metabolites from Omega‐3 fatty acids, has been reported to promote inflammation resolution in several inflammatory diseases. This study aims to investigate the effect of MaR1 on hypertensive vascular remodeling. Here, we found serum MaR1 levels were reduced in hypertensive patients and was negatively correlated with systolic blood pressure (SBP). The treatment of MaR1 reduced the elevation of blood pressure and alleviated vascular remodeling in the angiotensin II (AngII)‐infused mouse model. In addition, MaR1‐treated vascular smooth muscle cells (VSMCs) exhibited reduced excessive proliferation, migration, and phenotype switching, as well as impaired pyroptosis. However, the knockout of the receptor of MaR1, leucine‐rich repeat‐containing G protein‐coupled receptor 6 (LGR6), was seen to aggravate pathological vascular remodeling, which could not be reversed by additional MaR1 treatment. The mechanisms by which MaR1 regulates vascular remodeling through LGR6 involves the Ca^2+^/calmodulin‐dependent protein kinase II/nuclear factor erythroid 2‐related factor 2/heme oxygenase‐1 signaling pathway. Overall, supplementing MaR1 may be a novel therapeutic strategy for the prevention and treatment of hypertension.

## Graphical abstract

Maresin‐1 ameliorates VSMC proliferation, migration, phenotype transition, and pyroptosis through LGR6, thereby alleviating vascular remodeling in hypertension. The Ca^2+^/CaMKII/Nrf2/HO‐1 signaling pathway is involved in this process.

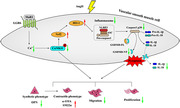

## 1 INTRODUCTION

Hypertension is one of the most notorious chronic diseases that leads to irreversible damage in important organs, including the cardiovascular system, brain, and kidney.^1^ It remains a huge problem in public, which accounts for approximately 30% of all deaths worldwide. Pathological vascular remodeling is not only a feature of hypertension, but also a pathological basis of maintaining hypertension.^2^ Reversing vascular remodeling may provide a new strategy for the management of hypertension. Vascular smooth muscular cells (VSMCs) comprise the majority of the cellular components in the aortic media and play a crucial role in vascular homeostasis.^3^, ^4^ In healthy vasculature, VSMCs exhibit a contractile phenotype that regulates blood pressure. Under pathological conditions, VSMCs might undergo phenotypic switching from a contractile phenotype to a synthetic phenotype, leading to excessive proliferation and migration, which is a core event of vascular remodeling.^5^

Evidence indicated that VSMC pyroptosis is also involved in pathological vascular remodeling.^6^ Inflammasomes and pyroptotic cell death are essential players for the progression of chronic vascular inflammation and damage. The NOD‐like receptor protein 3 (NLRP3) inflammasome is currently the best described inflammasome, consisting of a sensor NLRP3, an adapter apoptosis‐associated speck‐like protein containing a CARD (ASC), and an effector pro‐caspase‐1. After inflammasome assembly, pro‐caspase‐1 (p45) will be activated and cleaved into mature cleaved caspase‐1 (p20 subunit). Active caspase‐1 can recognize and process inactive precursors of IL‐1β and IL‐18 into their mature forms, and cleave the caustic executor protein GSDMD to into N‐terminal form (GSDMD‐N). In the vasculature, angiotensin II (AngII) triggers inflammasome activation, and then promotes the release of mature IL‐1β and IL‐18 through GSDMD‐cleaved pores in the plasma membrane, resulting in pyroptosis.^7^, ^8^

Omega‐3 fatty acids are polyunsaturated fatty acids and abundant in fish oil, which have potential benefits in several diseases.^9^ Maresin‐1 (MaR1) is one of the highlighted metabolites from Omega‐3 fatty acids that shows various strong anti‐inflammatory actions in inflammatory diseases.^10^ NLRP3 inflammasome‐induced pyroptosis can be inhibited by MaR1 delivery in acute liver injury,^11^ noncompressive lumbar disk herniation,^12^ and so on.^13^ To date, accumulating evidence reveal that MaR1 could mediate the resolution of acute inflammation as an endogenous mediator. However, the effect of MaR1 on the development of chronic diseases is unknown. Furthermore, although MaR1 has been identified in inflammation resolution, its conserved structure also functions in host defense, pain, organ protection and tissue remodeling.^14^ The role of MaR1 in vascular remodeling during hypertension needs to be investigated.

Considering the action mechanism of MaR1, a recent study identified one receptor, leucine‐rich repeat‐containing G‐protein‐coupled receptor 6 (LGR6), which has the structure of GPCRs and is widely identified in various tissues.^15^ MaR1 can activate LGR6 receptor, thereby promoting phagocyte immunoresolvent functions.^15^, ^16^ Furthermore, MaR1/LGR6 signaling can mitigate CXCL1 secretion and alleviate post‐lung transplant ischemia–reperfusion injury in mice.^17^ The MaR1/LGR6 axis maintains the balance between pulmonary artery smooth muscle cells (PASMCs) proliferation and apoptosis.^18^ In addition, LGR6 is required for MaR1 to regulate brown adipose tissue activation and white adipose tissue browning.^19^ However, the link between MaR1 and LGR6 in VSMCs under hypertensive condition remains unclear.

In this study, we investigated the effect of MaR1/LGR6 signaling on hypertensive vascular remodeling in an AngII‐induced hypertension mouse model and further analyzed its potential mechanisms. We tested the hypothesis that MaR1 might be a potential therapeutic option to attenuate hypertensive vascular damage.

## 2 RESULTS

### 2.1 MaR1 treatment alleviated hypertensive vascular remodeling in the AngII‐infused model

**FIGURE 1:**
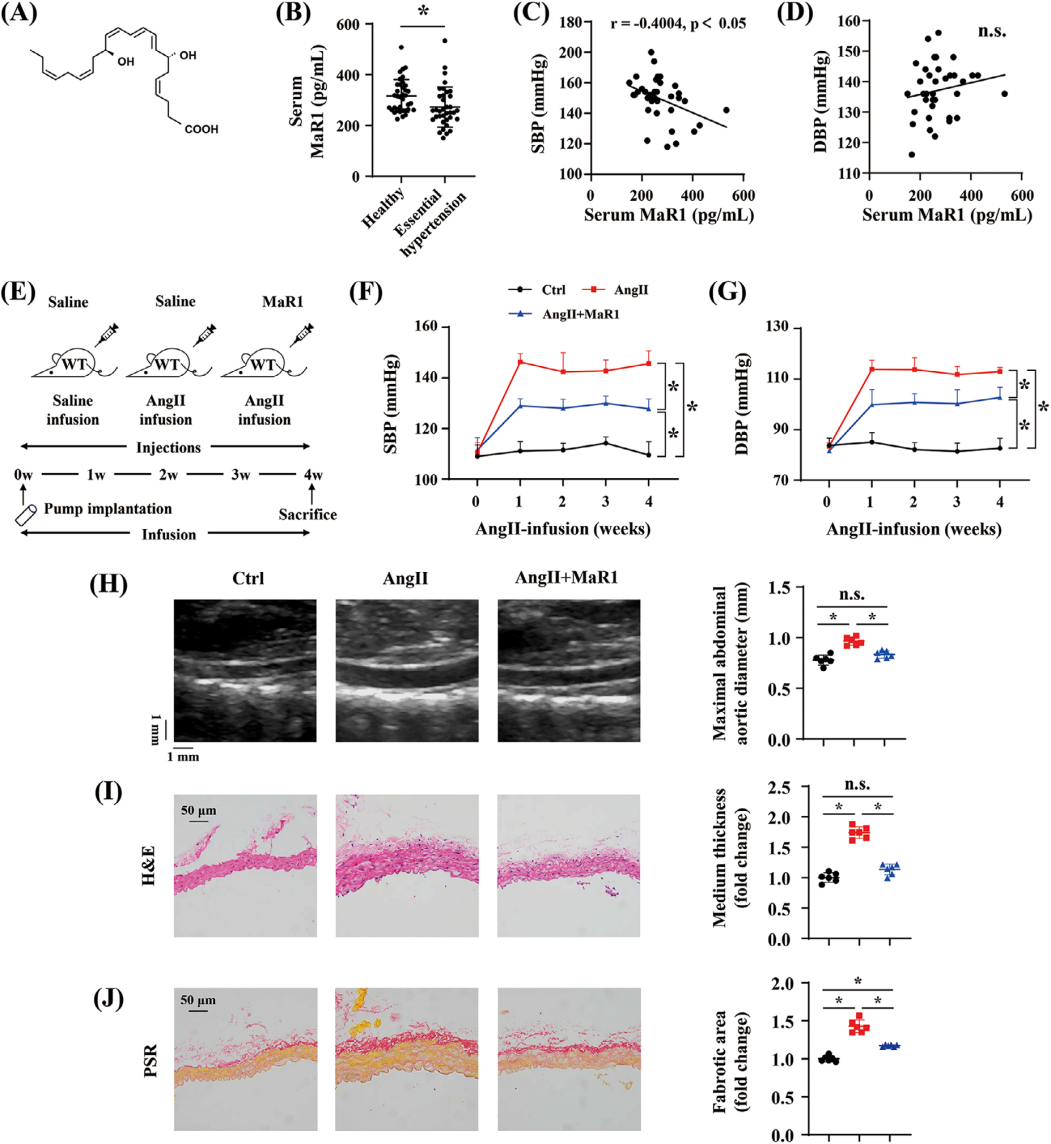
MaR1 treatment alleviated hypertensive vascular remodeling in the AngII‐infused model. (A) Chemical structure of Maresin‐1. (B) Human Maresin‐1 levels in serum samples from healthy controls (*n* = 35) and patients with hypertension (*n* = 35). *p < 0.05, Mann–Whitney *U* test. (C and D) Correlation analysis of systolic blood pressure (SBP), diastolic blood pressure (DBP), and MaR1 levels in hypertensive patients. *p < 0.05, Spearman's correlation analysis. (E) Procedure diagram. (F and G) Quantitative analysis of SBP and DBP at different time points during AngII infusion in Ctrl, AngII, and AngII+MaR1 groups (*n* = 6). ∗*p* < 0.05, two‐way ANOVA. (H) Representative ultrasound images of abdominal aorta and quantitation of maximal abdominal aortic diameter from the indicated groups (*n* = 6). ∗*p* < 0.05, one‐way ANOVA. (I) Representative hematoxylin and eosin (H&E) staining of abdominal artery cross‐sections and quantification of media thickness from the indicated groups (*n* = 6). ∗*p* < 0.05, one‐way ANOVA. (J) Representative images of picrosirius red (PSR) staining of abdominal artery cross‐sections and quantification of vascular fibrosis area from the indicated groups (*n* = 6). ∗*p* < 0.05, one‐way ANOVA.

MaR1 is a polyhydroxy and polyunsaturated conjugated double bond molecule metabolized from omega‐3 fatty acids (Figure 1A). To investigate the association between the MaR1 level and hypertension, we examined the serum of patients diagnosed with essential hypertension and compared it with healthy patients. Our findings revealed a decline in serum MaR1 levels in hypertensive patients (Figure 1B). Furthermore, we observed a negative correlation between MaR1 concentration and systolic blood pressure (SBP), but not with diastolic blood pressure (DBP) (Figures 1C and D). To further explore the role of MaR1 in hypertension, an AngII‐infused hypertension mouse model was used in our study (Figure 1E). Using a tail‐cuff system, we determined that there were no baseline differences in SBP and DBP among the groups. However, AngII infusion significantly elevated both SBP and DBP, whereas MaR1 treatment alleviated the AngII‐induced blood pressure elevation (Figures 1F and G). Additionally, after 28 days of AngII infusion, we assessed the maximal aortic diameter using abdominal ultrasonography. AngII infusion led to an increase in the abdominal aortic diameter, which was mitigated by MaR1 treatment (Figure 1H). Hematoxylin–eosin (H&E) staining demonstrated that MaR1 administration significantly reduced AngII‐induced medial thickening (Figure 1I). Moreover, picrosirius red (PSR) staining revealed a significant reduction in the vascular fibrosis area in the AngII‐infused mice following MaR1 administration (Figure 1J). These results suggested that MaR1 may attenuate hypertensive vascular remodeling in the AngII‐infused mouse model.

### 2.2 MaR1 treatment modulated VSMC phenotype transition and pyroptosis in the AngII‐infused model

**FIGURE 2:**
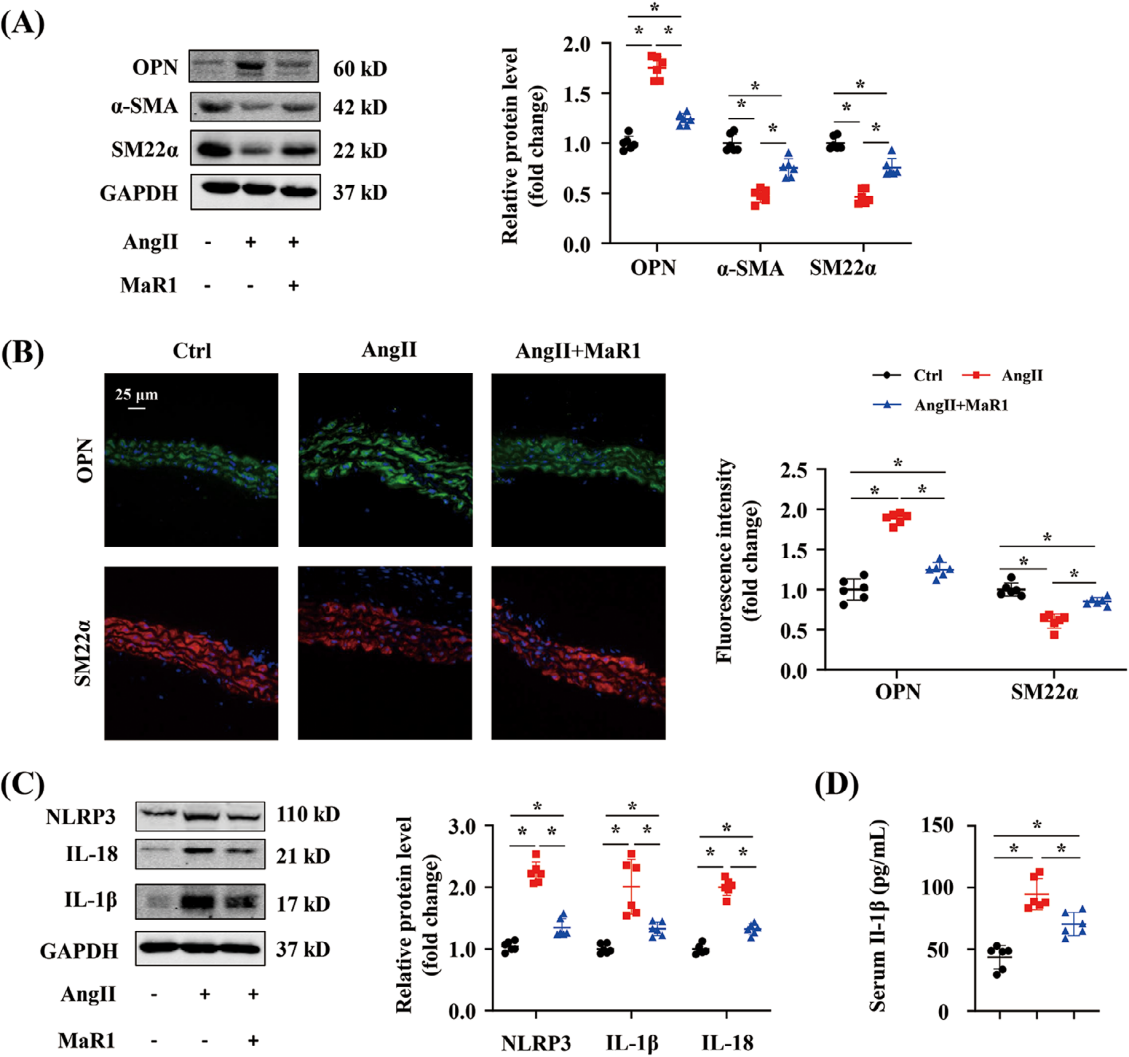
MaR1 treatment modulated VSMC phenotype transition and pyroptosis in the AngII‐infused model. (A) Immunoblotting analysis of α‐SMA, SM22α, and OPN in the aortas of Ctrl, AngII, and AngII+MaR1 groups (*n* = 6). ∗*p* < 0.05, one‐way ANOVA. (B) Representative images of SM22α and OPN immunofluorescence staining of abdominal artery cross‐sections from the indicated group (*n* = 6) and quantitative analysis. ∗*p* < 0.05, one‐way ANOVA. (C) Immunoblotting analysis of NLRP3, IL‐18, and IL‐1β in the aortas from the indicated group (*n* = 6). ∗*p* < 0.05, one‐way ANOVA. (D) Quantitative analysis of mouse IL‐1β levels in the serum from the indicated group (*n* = 6). ∗*p* < 0.05, one‐way ANOVA.

In hypertension, VSMC phenotypic transformation is marked by an increase in synthetic proteins such as osteopontin (OPN), and a decrease in contractile proteins such as α‐smooth muscle actin (α‐SMA) and smooth muscle 22α (SM22α).^20^ The synthetic protein OPN was upregulated, and contractile proteins α‐SMA and SM22α were downregulated in aortas after AngII infusion, whereas MaR1 administration significantly increased the protein levels of α‐SMA and SM22α and reduced OPN level (Figure 2A). Immunofluorescence staining further verified that MaR1 treatment reversed AngII‐induced undesirable changes in SM22α and OPN (Figure 2B). We also observed that the key protein, NLRP3, and the by‐products of pyroptosis, IL‐18 and IL‐1β, were increased in aortas of AngII‐infused mice, whereas MaR1 treatment reduced the expression of those proteins (Figure 2C). Enzyme‐linked immunosorbent assay (ELISA) testing showed that MaR1 administration allevaited the elevation of serum IL‐1β level in the AngII‐infused mice (Figure 2D).

### 2.3 LGR6 knockout with or without MaR1 treatment aggravated hypertensive vascular remodeling

**FIGURE 3:**
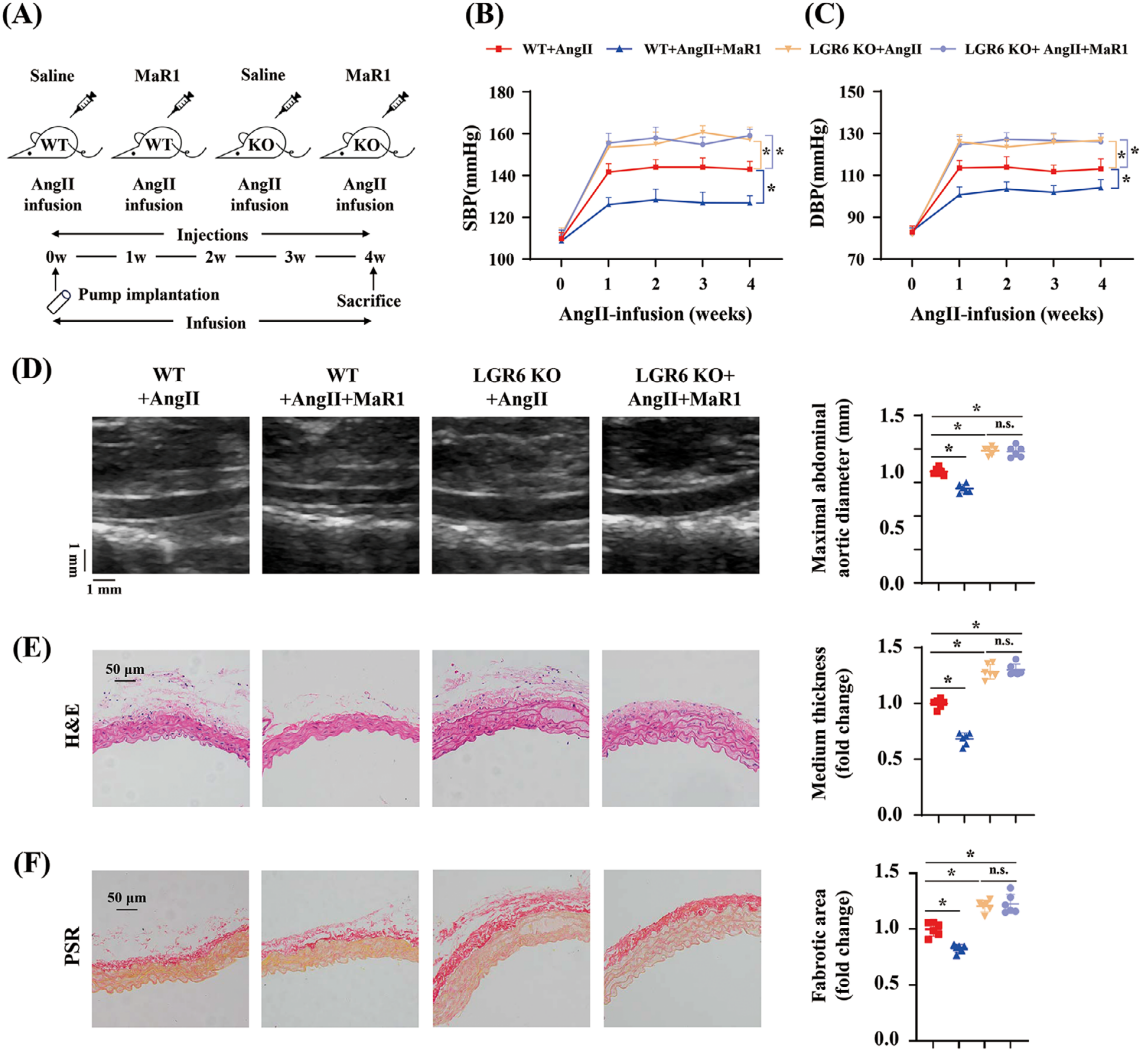
LGR6 knockout with or without MaR1 treatment aggravated hypertensive vascular remodeling in the AngII‐infused model. (A) Procedure diagram. (B and C) Quantitative analysis of SBP and DBP at different time points during AngII infusion in WT or LGR6 KO mice with or without MaR1 treatment (*n* = 6). ∗*p* < 0.05, two‐way ANOVA. (D) Representative ultrasound images of abdominal aorta and quantitation of maximal abdominal aortic diameter from WT+AngII, WT+AngII+MaR1, LGR6 KO+AngII, and LGR6 KO+AngII+MaR1 groups (*n* = 6). ∗*p* < 0.05, one‐way ANOVA. (E) Representative H&E staining of abdominal artery cross‐sections and quantification of media thickness from the indicated groups (*n* = 6). ∗*p* < 0.05, one‐way ANOVA. (F) Representative images of PSR staining of abdominal artery cross‐sections and quantification of vascular fibrosis area from the indicated groups (*n* = 6). ∗*p* < 0.05, one‐way ANOVA.

To investigate the role of the receptor LGR6 in pathological hypertension, we generated LGR6 knockout (KO) mice (Figure S1A) and treated them with AngII (Figure 3A). Blood pressure measurements showed that LGR6 KO aggravated the elevation of SBP and DBP, which were not reversed by MaR1 treatment (Figures 3B and C). Vascular ultrasound images and maximal aortic diameter measurement at day 28 after AngII infusion demonstrated that LGR6 KO in the presence or absence of MaR1 treatment aggravated AngII‐induced aortic dilation (Figure 3D). An increase in aortic medial thickness and vascular fibrosis area was observed in AngII‐infused LGR6 KO mice with or without MaR1 treatment compared with AngII‐infused wild‐type (WT) mice (Figures 3E and F).

**FIGURE 4:**
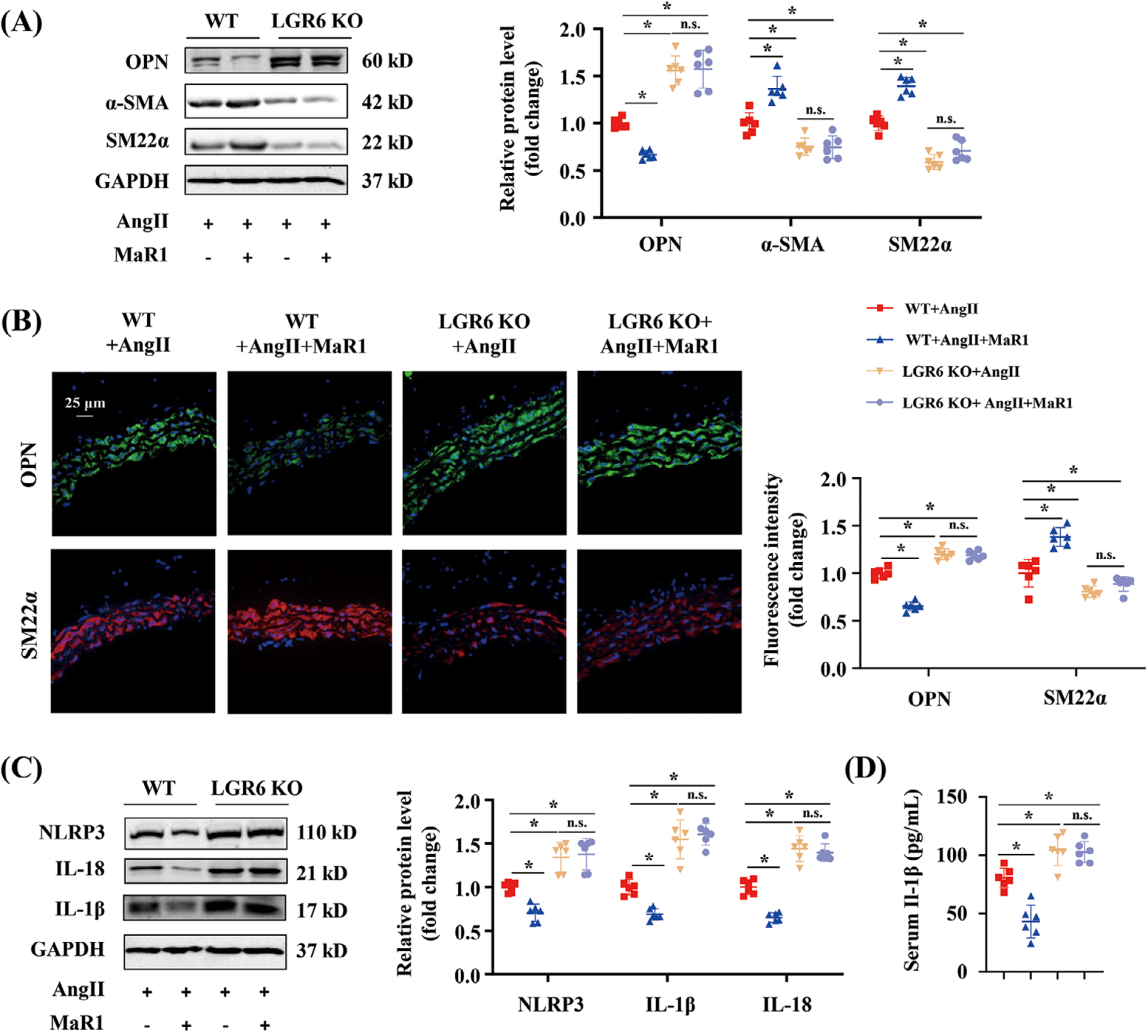
LGR6 knockout with or without MaR1 treatment aggravated VSMC phenotype transition and pyroptosis in the AngII‐infused model. (A) Immunoblotting analysis of α‐SMA, SM22α, and OPN protein levels in the aortas from WT+AngII, WT+AngII+MaR1, LGR6 KO+AngII, and LGR6 KO+AngII+MaR1 groups. ∗*p* < 0.05, one‐way ANOVA. (B) Representative images of SM22α and OPN immunofluorescence staining of abdominal artery cross‐sections from the indicated groups (*n* = 6) and quantitative analysis. ∗*p* < 0.05, one‐way ANOVA. (C) Immunoblotting analysis of NLRP3, IL‐18, and IL‐1β in the aortas from the indicated groups. ∗*p* < 0.05, one‐way ANOVA. (D) Quantitative analysis of mouse IL‐1β levels in the serum from the indicated groups (*n* = 6). ∗*p* < 0.05, one‐way ANOVA.

Western blot showed that the synthetic protein OPN was upregulated, and contractile proteins α‐SMA and SM22α were downregulated in aortas from AngII‐infused LGR6 KO mice, and these changes were not affected by MaR1 treatment (Figure 4A). Immunofluorescence staining further verified that LGR6 KO with or without MaR1 treatment promoted undesirable changes in SM22α and OPN (Figure 4B). Moreover, AngII‐infused LGR6 KO mice exhibited an increase in the aortic level of NLRP3, IL‐18, and IL‐1β, and the serum IL‐1β level, which could not be rescued by MaR1 administration (Figures 4C and D). These results indicated that LGR6 KO aggravated hypertensive vascular remodeling in the AngII‐infused model, which could not be reversed by MaR1 treatment.

### 2.4 MaR1 regulated VSMC proliferation, migration, phenotype transition, and pyroptosis through its receptor LGR6

**FIGURE 5:**
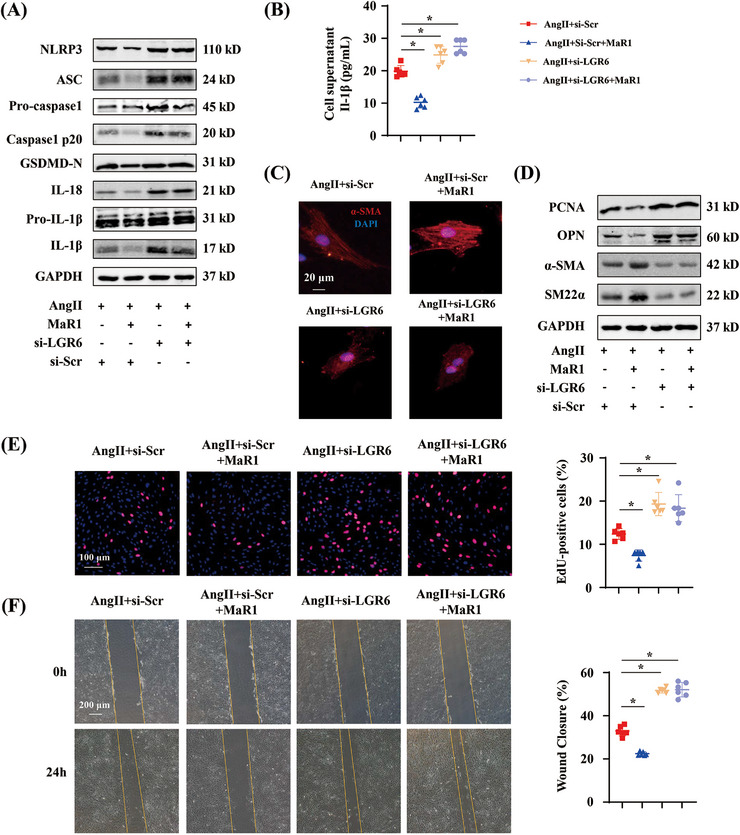
MaR1 regulated VSMC proliferation, migration, phenotype transition, and pyroptosis through its receptor LGR6. (A) Immunoblotting analysis of NLRP3, ASC, pro‐caspase‐1, caspase‐1 p20, GSDMD‐N, IL‐18, pro‐IL‐1β, and IL‐1β in AngII‐treated RASMCs transfected by siRNA in the presence or absence of MaR1 (100 nM) for 24 h (*n* = 6). (B) Quantitative analysis of Il‐1β content in RASMC culture supernatant from the indicated groups (*n* = 6). *p < 0.05, one‐way ANOVA. (C) Representative images of α‐SMA immunofluorescence staining of RASMCs from the indicated groups. (D) Immunoblotting analysis of PCNA, OPN, α‐SMA, and SM22α in RASMCs from the indicated groups (*n* = 6). (E) Representative images of EdU staining of RASMCs from the indicated groups (*n* = 6) and quantitative analysis. ∗*p* < 0.05, one‐way ANOVA. (F) Representative images of wound healing of RASMCs from the indicated groups (*n* = 6) and quantitative analysis. ∗*p* < 0.05, one‐way ANOVA.

To gain insight into the underlying mechanism of the MaR1/LGR6 axis in hypertensive mice, we conducted in vitro experiments using rat aortic smooth muscle cells (RASMCs). Western blot showed that MaR1‐treated RASMCs exhibited a decrease in the levels of NLRP3, ASC, caspase‐1 p20, IL‐1β, IL‐18, and GSDMD‐N. Conversely, LGR6 deficiency in the presence or absence of MaR1 treatment led to an increase in these protein levels (Figure 5A). ELISA testing further verified that IL‐1β level was elevated in the culture medium of LGR6 knockdown RASMCs, regardless of MaR1 treatment (Figure 5B). Immunofluorescence showed that the RASMCs treated with MaR1 showed high levels of α‐SMA and myofilament content, whereas LGR6 deficiency reversed those changes (Figure 5C). A decrease in proliferating cell nuclear antigen (PCNA) and OPN and an increase in α‐SMA and SM22α were observed in MaR1‐treated RASMCs, whereas LGR6 deficiency in the presence or absence of MaR1 treatment produced the opposite result (Figure 5D). Furthermore, LGR knockdown with or without MaR1 treatment enhanced the excessive proliferation of RASMCs, as illustrated by the 5‐ethynyl‐2′‐deoxyuridine (EdU) assay (Figure 5E). We further examined the effect of the MaR1/LGR6 axis on RASMC migration using a wound healing assay. At the 24‐h timepoint, MaR1 significantly inhibited excessive RASMC migration, whereas LGR6 deficiency with or without MaR1 treatment produced the opposite effect (Figure 5F). Together, these results demonstrated that the MaR1/LGR6 axis mediated VSMC proliferation, migration, phenotype transition, and pyroptosis during AngII stimulation.

### 2.5 LGR6 KO mediates vascular biologic function through promoting Ca^2+^ influx and phosphorylation of calmodulin‐dependent protein kinase II

**FIGURE 6:**
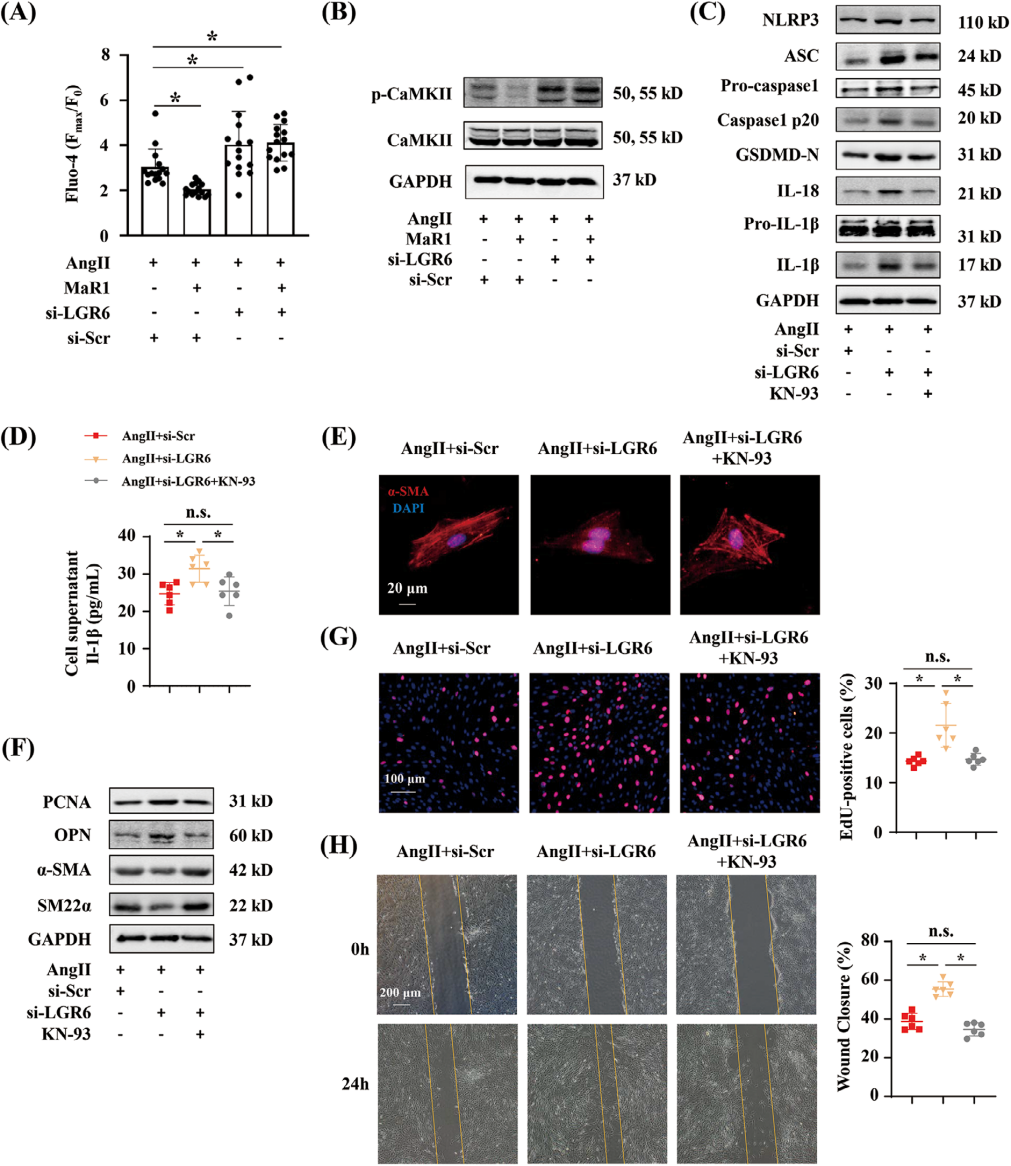
LGR6 knockout mediates vascular biologic function through promoting Ca^2+^ influx and phosphorylation of calmodulin‐dependent protein kinase II (CaMKII). (A) Quantitative analysis of *F*_max_/*F*_0_ (*F*_max_: maximum fluorescence intensity upon stimulation; *F*_0_: mean fluorescence intensity before stimulation) ratios in RASMCs transfected by siRNA in the presence or absence of MaR1 (100 nM) (*n* = 15). Cells were stimulated with 1000 nmol/L AngII. ∗*p* < 0.05, one‐way ANOVA. (B) Immunoblotting analysis of p‐CaMKII and CaMKII in RASMCs transfected by siRNA in the presence or absence of MaR1 (100 nM) for 24 h (*n* = 6). (C) Immunoblotting analysis of NLRP3, ASC, pro‐caspase‐1, caspase‐1 p20, GSDMD‐N, IL‐18, pro‐IL‐1β, and IL‐1β in RASMCs transfected by siRNA in the presence or absence of KN‐93 (20 μM) for 24 h (*n* = 6). (D) Quantitative analysis of Il‐1β content in RASMC culture supernatant from the indicated groups (*n* = 6). *p < 0.05, one‐way ANOVA. (E) Representative images of α‐SMA immunofluorescence staining of RASMCs from the indicated groups. (F) Immunoblotting analysis of PCNA, OPN, α‐SMA, and SM22α in RASMCs from the indicated groups (*n* = 6). (G) Representative images of EdU staining of RASMCs from the indicated groups (*n* = 6) and quantitative analysis. ∗*p* < 0.05, one‐way ANOVA. (H) Representative images of wound healing of RASMCs from the indicated groups (*n* = 6) and quantitative analysis. ∗*p* < 0.05, one‐way ANOVA.

As we all know, G protein‐coupled receptor is associated with intracellular Ca^2+^. Evidence indicated that MaR1 could regulate the intracellular level of Ca^2+^ in conjunctival goblet cells and DRG neurons.^21^, ^22^ We assumed that MaR1 could alter the intracellular Ca^2+^ of VSMCs through LGR6. Next, we measured the level of intracellular Ca^2+^ in RASMCs when stimulated by AngII. By using Fluo‐4 AM, we found that MaR1 treatment reduced AngII‐induced intracellular Ca^2+^ elevation in RASMCs, whereas the elevation of intracellular Ca^2+^ was enhanced by LGR6 deficiency in the presence or absence of MaR1 treatment (Figure 6A). Calmodulin‐dependent protein kinase II (CaMKII) is a downstream target of Ca^2+^. Western blot showed that MaR1 treatment reduced the phosphorylation of CaMKII in AngII‐treated RASMCs, whereas LGR6 deficiency with or without MaR1 treatment produced the opposite result (Figure 6B). For further investigation, we treated LGR6‐defective RASMCs with a CaMKII inhibitor, KN‐93. Western blot and ELISA showed that KN‐93 treatment reversed the propyroptotic effect of LGR6 deficiency in AngII‐treated RASMCs (Figures 6C and D). Moreover, KN‐93 largely reversed the role of LGR6 deficiency in promoting RASMC phenotype transition (Figures 6E and F). The EdU assay further indicated that KN‐93 reversed the proproliferation effect of LGR6 deficiency (Figure 6G). Wound healing assay showed that excessive migration triggered by LGR6 deficiency was suppressed by KN‐93 (Figure 6H). These results demonstrated that LGR6/Ca^2+^/CaMKII pathway regulated VSMC proliferation, migration, phenotype transition, and pyroptosis.

### 2.6 Nuclear factor E2‐related factor 2/heme oxygenase‐1 pathway serves as the downstream of the MaR1/LGR6/Ca^2+^/CaMKII axis to regulate VSMC biologic function and pyroptosis

**FIGURE 7:**
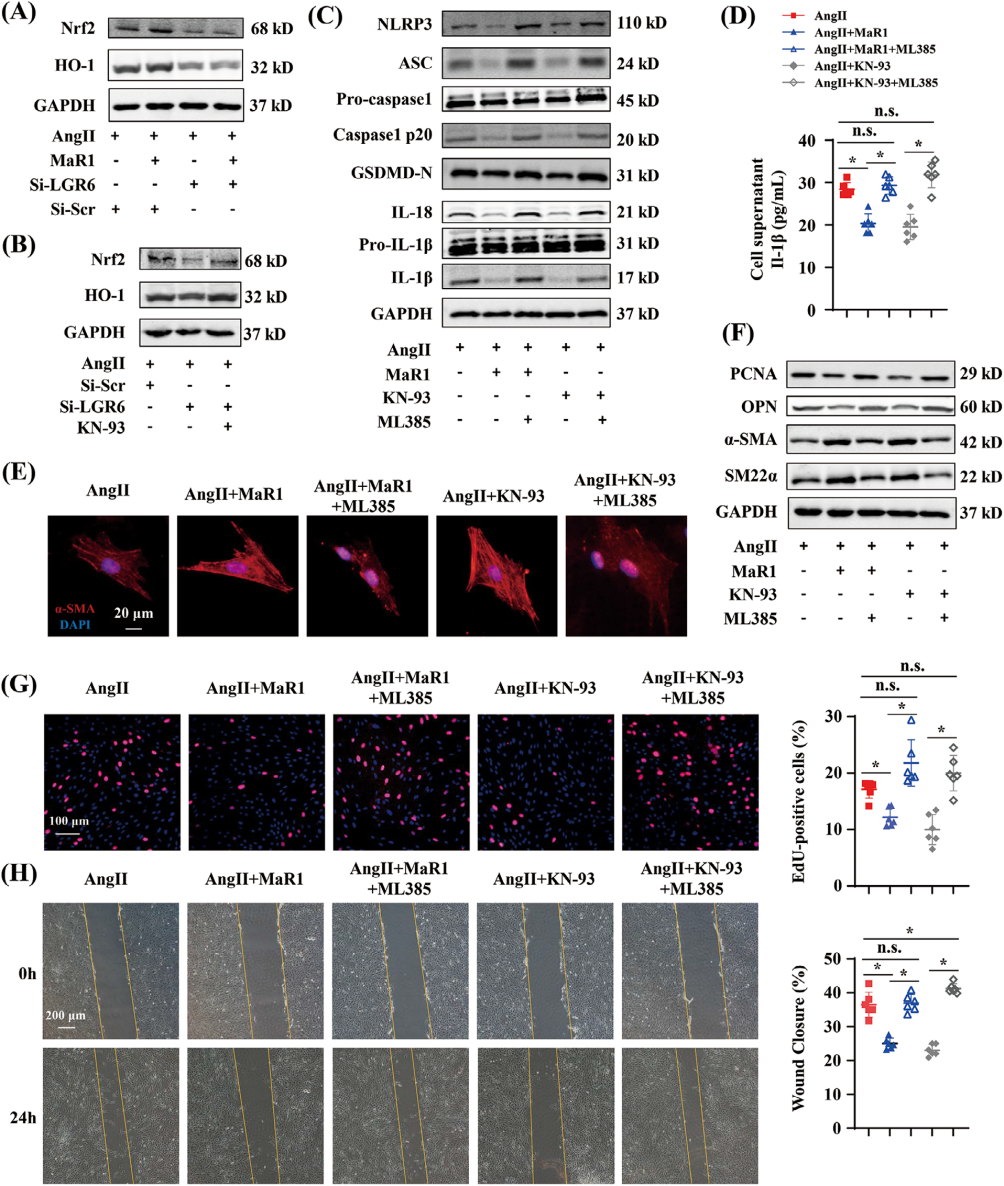
Nrf2/HO‐1 pathway serves as the downstream of the MaR1/LGR6/Ca^2+^/CaMKII axis to regulate VSMC biologic function and pyroptosis. (A) Immunoblotting analysis of Nrf2 and HO‐1 in AngII‐treated RASMCs transfected by siRNA in the presence or absence of MaR1 (100 nM) for 24 h (*n* = 6). (B) Immunoblotting analysis of Nrf2 and HO‐1 in AngII‐treated RASMCs transfected by siRNA in the presence or absence of KN‐93 (20 μM) for 24 h (*n* = 6). (C) Immunoblotting analysis of NLRP3, ASC, pro‐caspase‐1, caspase‐1 p20, GSDMD‐N, IL‐18, pro‐IL‐1β, and IL‐1β in RASMCs transfected by siRNA in the presence or absence of MaR1(100 nM), KN‐93 (20 μM), or ML385 (5 μM) for 24 h (*n* = 6). (D) Quantitative analysis of Il‐1β content in RASMC culture supernatant from the indicated groups (*n* = 6). *p < 0.05, one‐way ANOVA. (E) Representative images of α‐SMA immunofluorescence staining of RASMCs from the indicated groups. (F) Immunoblotting analysis of PCNA, OPN, α‐SMA, and SM22α in RASMCs from the indicated groups (*n* = 6). (G) Representative images of EdU staining of RASMCs from the indicated groups (*n* = 6) and quantitative analysis. ∗*p* < 0.05, one‐way ANOVA. (H) Representative images of wound healing of RASMCs from the indicated groups (*n* = 6) and quantitative analysis. ∗*p* < 0.05, one‐way ANOVA.

Previous studies have shown that the nuclear factor E2‐related factor 2 (Nrf2) plays a critical role in cytoprotection and is associated with inflammasome activation and pyroptotic cell death.^23^, ^24^ Heme oxygenase‐1 (HO‐1) is the downstream of Nrf‐2. Evidence suggested that phosphorylation of CaMKII activated Nrf2/HO‐1 signaling pathway. Western blot showed that the protein levels of Nrf2 and HO‐1 in RASMCs were increased by MaR1 treatment, while LGR6 deficiency with or without MaR1 treatment downregulated those protein levels (Figure 7A). Moreover, KN‐93 reversed the decreased levels of Nrf2 and HO‐1 induced by LGR6 deficiency (Figure 7B). Hence, we assumed that Nrf2/HO‐1 signaling pathway is the downstream target of the MaR1/LGR6/Ca^2+^/CaMKII signaling pathway. To prove our hypothesis, we used a Nrf2 inhibitor, ML385, as an intervention in our study. Western blot showed that MaR1 or KN‐93 decreased protein levels of NLRP3, ASC and caspase‐1 p20, IL‐1β, IL‐18, and GSDMD‐N, which was reversed by ML385 (Figure 7C). ELISA testing showed that ML385 attenuated the inhibitory effect of MaR1 or KN‐93 on IL‐1β generation (Figure 7D). Western blot showed that MaR1 or KN‐93 inhibited the phenotype transition of RASMCs, whereas ML385 promoted this process (Figures 7E and F). The EdU assay and wound healing assay showed that ML385 treatment abolished the antiproliferative and antimigratable effects of MaR1 or KN‐93 (Figures 7G and H). In summary, our findings suggested that Nrf2/HO‐1 signaling pathway was the downstream of MaR1/LGR6/Ca^2+^/CaMKII axis.

Next, to further confirm that MaR1 could serve as an activator of Nrf2 in RASMCs, we compared the effects of MaR1 and NK‐252, a Nrf2 activator. In LGR6‐defective RASMCs, it was NK‐252, not MaR1 that exerted its antipyroptotic effect (Figures S2A and B). Immunofluorescence and western blot showed that NK‐252 inhibited phenotype switching of LGR6‐defective RASMCs, while MaR1 cannot (Figures S2C and D). In addition, NK‐252 has no effect on the phosphorylation of CaMKII (Figure S2E), which verified Nrf2/HO‐1 pathway serving as the downstream of the Ca^2+^/CaMKII axis. The excessive proliferation and migration ability of LGR6‐defective RASMCs was impaired by the treatment with NK‐25 instead of MaR1 (Figures S2F and G). These results suggested that MaR1 serving as a Nrf2 activator in AngII‐treated RASMCs required the presence of its receptor LGR6.

## 3 DISCUSSION

In this study, we found that the MaR1/LGR6 axis plays a crucial role in hypertensive vascular remodeling. Our data suggested that the circulatory MaR1 level was reduced in patients with hypertension. MaR1 treatment reduced blood pressure elevation and alleviated hypertensive vascular remodeling in the AngII‐infused mice, whereas LGR6 deficiency in the presence or absence of MaR1 treatment produced the opposite results. The mechanism involves Ca^2+^/CaMKII/Nrf2/HO‐1 pathway. Our findings suggest that MaR1 alleviates hypertension, which provides new insight into the treatment of hypertension.

MaR1 is a member of specialized proresolving lipid mediators (SPMs) derived from polyunsaturated fatty acids. Lipoxins, resolvins, protectins, and maresins are four structurally unique different families of SPMs.^25^ The concentration of SPMs in the serum may give a hint of the severity of the disease. Endogenous serum MaR1 was decreased in patients with pulmonary arterial hypertension (PAH) and experimental PAH mice.^18^ The MaR1 concentration was higher in the patients with inactive rheumatoid arthritis (RA) and lower in the patients with active RA.^26^ Additionally, the decreased serum MaR1 level was associated with postmenopausal osteoporosis.^27^ The serum level of MaR1 was significantly reduced in type 2 diabetic patients compared with normal participants, particularly in patients with diabetic foot ulcer or diabetic kidney disease.^28^ The decreased serum MaR1 concentration tightly associated with obesity, impaired glucose and lipid metabolism, reduced first‐phase of glucose‐stimulated insulin secretion, and enhanced insulin resistance.^29^ Our study revealed that the serum level of MaR1 was reduced in patients with hypertension compared with healthy controls. In addition, the serum level was negatively correlated to the value of SBP in hypertensive patients. These results indicated that the decreased MaR1 level might be a warning for hypertension.

MaR1 favors directly mediating the function of macrophages in the regulation of vascular diseases.^30^ In the development of atherosclerosis, MaR1 delivery induced a shift in macrophage profile toward a reparative phenotype, helping to create a homeostasis‐restoring environment for plaque stability.^31^ Moreover, MaR1 treatment suppressed murine abdominal aortic aneurysm formation through promoting macrophage‐dependent efferocytosis of apoptotic and necrotic cells.^16^ In addition to macrophages, VSMCs, the major cell type of medial layer, play a crucial role in the development of vascular diseases. In response to vascular stress, such as AngII, viable VSMCs are prone to differentiate, including proliferation, migration, and phenotype transition.^2^ Furthermore, hypertensive stress also activates inflammasome and triggers pyroptosis in VSMCs, which contributes to vascular remodeling.^32^, ^33^ GSDMD deficiency in VSMCs ameliorates vascular remodeling in abdominal aortic aneurysm.^34^ Our study demonstrated that MaR1 could inhabit VSMC differentiation and pyroptosis induction, thereby reversing hypertensive vascular remodeling.

LGR6, the receptor of MaR1, is identified to be a type of G protein‐coupled receptors. The role of G protein‐coupled receptors in the development of hypertension varies by type. GPR75 is required for 20‐hydroxyeicosatetraenoic acid‐dependent hypertension, and GPR75 knockdown prevented blood pressure elevation, endothelial dysfunction, and vascular remodeling.^35^ The cardioprotective effect of short‐chain fatty acids was mediated via the cognate receptors GPR43/GPR109A.^36^ Prostaglandin D2 receptor 1, as a G protein‐coupled receptor, protects against VSMC transition to myofibroblasts in AngII‐induced hypertension in mice.^37^ In this study, LGR6 is required for MaR1‐mediated intracellular Ca^2+^ decrease in VSMCs under AngII stress. Evidence showed that Ca^2+^/CaMKII signaling in VSMCs could mediate the excessive proliferation, migration, and differentiation.^38^, ^39^ CaMKII inhibition in VSMCs reduced aortic remodeling and AngII‐induced hypertension.^40^ Our data indicated that impaired MaR1/LGR6 axis promoted vascular remodeling through Ca^2+^/CaMKII signaling. Another study revealed that MaR1 reversed abnormal changes in pulmonary vascular remodeling in a murine model of PAH. This study indicated that MaR1/LGR6 axis inhibited PASMC proliferation and migration and promoted cell apoptosis through decreased hypoxia‐induced phosphorylation of STAT3, AKT, ERK, and FoxO1. Additionally, endogenous serum MaR1 was decreased in PAH patients, which is consistent with our conclusion.^18^

Nrf2 and its downstream HO‐1 are regulators of the antioxidant system. Evidence demonstrated that Nrf2/HO‐1 signaling could regulate antioxidation and anti‐inflammation via directly inhibiting cytokines storm, inflammasome activation, and pyroptosis.^41^ The activation of Nrf2/HO‐1 signaling protected against doxorubicin‐induced cardiotoxicity through upregulation of NLRP3 inflammasome activation and GSDMD‐mediated pyroptosis.^42^, ^43^ Knockout of Nrf2 abolished Syringaresinol‐mediated renoprotection and antipyroptotic effect in diabetic mice. Our study showed that MaR1 partially acts as a Nrf2 activator in the regulation of pyroptosis, and thus reverses hypertensive vascular remodeling.

Our study demonstrates the effect of MaR1 and its receptor LGR6 during the progression of hypertension. MaR1 activates LGR6 in VSMCs, blocking intracellular Ca^2+^ elevation, leading to VSMC dedifferentiation and pyroptosis inhibition, therefore alleviating vascular remodeling and hypertension progression. Exogenous MaR1 supplement could be a promising therapy for the treatment of hypertension.

## 4 MATERIALS AND METHODS

### 4.1 Studied patients

Consecutive 36 patients with were recruited in physical examination center, Renmin Hospital of Wuhan University. Inclusion criteria: SBP ≥ 140 mmHg or DBP ≥ 90 mmHg, patients with essential hypertension were first found within a month without treatment, or patients with essential hypertension did not receive effective treatment and still meet the above criteria; 18 years old ≤ age ≤ 80 years old. Exclusion criteria: concomitant disease, including cardiovascular disease, diabetes mellitus, autoimmune disease, hepatic disease, kidney disease, and so on; gestation period. In addition, 36 age‐sex matched healthy controls were recruited in the same period. Blood samples were obtained and serum samples were prepared, as described in our previous study.^44^

### 4.2 Reagent

Maresin‐1 and Maresin‐1 ELISA kit were purchased from Cayman Chemical. AngII was purchased from Enzo Life Sciences. Mouse IL‐1β ELISA kit was purchased from Neobioscience. EdU imaging kits was purchased from APExBIO. ML385 and KN‐93 were purchase from Topscience. Fluo‐4 AM was purchased from Invitrogen. Fetal bovine serum was purchased from Inner Mongolia Opcel Biotechnology Co., Ltd. Primary antibodies included: anti‐GAPDH (Abcolonal), anti‐SM22α (Servicebio), anti‐ASC (Santa Cruz Biotechnology), anti‐caspase‐1 p20 (Santa Cruz Biotechnology), anti‐IL‐1β (Santa Cruz Biotechnology), anti‐OPN (Immunoway), anti‐NLRP3 (Immunoway), anti‐IL‐18 (ImmunoWay), anti‐GSDMD‐N (Immunoway), anti‐Nrf2 (GeneTex), anti‐α‐SMA(Abcam), anti‐HO‐1(Abcam), anti‐CaMKII (Abcam), anti‐p‐CaMKII (Zenbio), anti‐PCNA (Cell Signaling Technology), anti‐rabbit IgG (H+L) (Cell Signaling Technology), anti‐mouse IgG (H+L) (Cell Signaling Technology), Cy3‐conjugated goat anti‐rabbit IgG (H+L) (Servicebio), and Alexa Fluor® 488‐conjugated goat anti‐rabbit IgG (H+L) (Servicebio).

### 4.3 Animals and experimental models

Mice used in the experiment were treated in accordance with the National Institute of Health Guidelines for the Care and Use of Laboratory Animals, and the study was approved by Wuhan University Animal Research Ethics Committee. 10‐week‐old C57BL/6J mice were purchased from Gempharmatech Co., Ltd. The heterozygous LGR6 KO mice with a C57BL/6 background were generated by Gempharmatech Co., Ltd. The homozygous mice were produced by intercrossing heterozygous mice. The mice were maintained in a standard laboratory at the Cardiovascular Research Institute of Wuhan University with a standard humidity/temperature‐controlled environment (70% relative humidity, 22°C) in a light‐controlled room (a 12:12 h light‐dark cycle) with access to sterile rodent food and water.

Mice were anesthetized with 2% isoflurane prior to surgery. Mice at 10−12 weeks of age were used in an AngII‐induced hypertension model by subcutaneous infusion of AngII at a dose of 750 ng/kg/min or saline using osmotic mini‐pumps (Alzet Model 2004) for 28 days, as described previously.^45^ We started the treatment of MaR1 or saline at the same time as the osmotic mini‐pumps implanted. For the group with MaR1 treatment, mice received an intraperitoneal injection of MaR1 at a dose of 2 μg/kg for 28 days. For the control group, mice were treated with saline. At the end of chronic infusion, mice were euthanized by CO_2_ inhalation, and their aortas were isolated for subsequent experiments.

### 4.4 Enzyme‐linked immunosorbent assay

Circulatory MaR1 was assayed using a Maresin‐1 ELISA kit. Circulation and supernatant IL‐1β was assayed using a mouse IL‐1β ELISA kit. Procedures were executed in accordance with the manufacturer's instructions.

### 4.5 Blood pressure measurement

The SBP and DBP of each mouse were measured by using a tail‐cuff system (CODA, Kent Scientific) at different time points after AngII or saline infusion as previously described.^46^ Briefly, a noninvasive tail‐cuff system was assembled, and the heating plate was preheated to approximately 37°C. The fixator was used to fix the position of the mouse. The head of the mouse was placed in the nose fixator, and the tail was placed through a hole in the back fixator. Then, the entire fixator was placed on the heating plate to keep mice warm. The pressurized tail sleeve was placed on the mouse's tail as close as possible to the tail root, and the sensor was positioned near the pressurized tail sleeve. Then, the program was started, and measurements were obtained and recorded.

### 4.6 Ultrasonic imaging

Ultrasonic images of the abdominal aorta were obtained using a small animal ultrasound system Vevo 2100 apparatus (Visual Sonics). The maximum abdominal aortic diameters were also examined using the Vevo 2100 apparatus. Each mouse was anesthetized with 2% isoflurane during the ultrasonography process.

### 4.7 Histological analysis

Aorta specimens were obtained immediately after sacrifice and fixed in 10% formalin for 48 h before being dehydrated and embedded in paraffin. The specimens were then cut transversely into 5 μm sections and stained with H&E and PSR as described previously.^47^ For immunofluorescence analysis, sections were incubated with primary antibodies overnight at 4°C. All images were obtained with a fluorescence microscope (Olympus Dx51, Japan), and were analyzed using Image‐J software.

### 4.8 Western blot analysis

Total protein was extracted from aorta tissues using RIPA buffer and quantified using a BCA protein assay kit, as described previously.^48^ Proteins (50 μg per sample) were separated by SDS‐PAGE gel electrophoresis and transferred to PVDF membranes which were then incubated with primary antibodies overnight at 4°C before being incubated with secondary antibodies. Membranes were visualized in an Odyssey infrared imaging system (LI‐COR, USA) and protein expression levels were normalized to that of GAPDH.

### 4.9 Cell isolation and culture

RASMCs were isolated from normal rat aortas, as described previously.^49^ The RASMCs were cultured routinely in DMEM medium containing 10% fetal bovine serum, supplemented with penicillin (100 U/mL) and streptomycin (100 μg/mL) at 37°C with a humidified atmosphere of 5% CO_2_. The primary RASMCs were identified using anti‐α‐SMA antibody. For all experiments, RASMCs (2–5 passages) were used following by quiescence for 12 h. The dose of AngII stimulus in vitro was 1000 nM.

### 4.10 siRNA transfection

For establishing the cells with LGR6 knockdown, siRNA against rat LGR6 and scramble siRNA were synthesized by Sangon Biotech. RASMCs were transfected with siRNA (20 nM) using Lipofectamine RNAiMAX (Thermo Fisher Scientific).

### 4.11 Monitoring of Ca^2+^ measurement in live RASMCs

RASMCs were loaded with 10 μmol/L Fluo‐4 AM, observed with Leica AF6000 fluorescence microscope, and Ca^2+^ handling detected as previously described. Briefly, Ca^2+^ transient was recorded under AngII stimulation.

### 4.12 EdU incorporation assay

RASMC proliferation was further with EdU imaging kits (Cy3) following the manufacturer's protocol. Cells were incubated with 50 mM EdU solution at 37°C for 2 h, fixed with 3.7% paraformaldehyde and permeabilizated, and then stained with Apollo Dye reaction and Hoechst stain. The EdU‐positive cells were counted and normalized by the total number of Hoechst 33342‐stained cells. Finally, cells were observed and imaged using a fluorescence microscope.

### 4.13 Wound healing assay

RASMCs were seeded into six‐well plates and incubated for 24 h. The wound was scratched with a pipette tip and washed with phosphate buffered saline to remove floating cells in the medium. VSMCs were treated with stimulus for 24 h, after which they were examined using a light microscope. The wound healing area was measured using Image‐J software.

### 4.14 Statistics

All the values were presented as the means ± standard deviations. Two‐group comparisons were performed using unpaired two‐tailed Student's *t*‐test for data with normal distribution, otherwise, nonparametric Mann–Whitney *U* test was used for data with non‐normal distribution or unequal variances. Multiple groups comparisons were assessed with one‐way analysis of variance (ANOVA) followed by Tukey's test. For experiments with two factors, two‐way ANOVA followed by Tukey's test. *p* Values of less than 0.05 were considered significant.

## AUTHOR CONTRIBUTIONS

*Conceptualization*: Zheng Yin and Menglong Wang. *Data curation*: Shanshan Peng, Shuwan Xu, and Cheng Wei. *Formal analysis*: Jishou Zhang and Shanshan Peng. *Investigation*: Zheng Yin and Shanshan Peng. *Methodology*: Jianfang Liu, Mengmeng Zhao, and Jun Wan. *Project administration*: Yao Xu and Jun Wan. *Software*: Zheng Yin, Jishou Zhang, and Juan‐juan Qin. *Supervision*: Jing Ye, Jun Wan, and Menglong Wang. *Writing‐original draft*: Zheng Yin and Jishou Zhang. All authors have read and approved the final manuscript.

## CONFLICT OF INTEREST STATEMENT

The authors declare that they have no conflict of interest.

## ETHICS STATEMENT

Animal and clinical experiments were approved by the Ethics Committee of Renmin Hospital of Wuhan University (Approval Number: WDRM20210703B, 2022K‐K198).

## Supporting information

Supporting information

## Data Availability

All data generated or analyzed in this study are available from the corresponding author on reasonable request.
