# Supplementary material for: Maresin‐1 ameliorates hypertensive vascular remodeling through its receptor LGR6
Source: MedComm (2020). 2024 Mar 9;5(3):e491. doi: 10.1002/mco2.491 (PMC10924638; doi:10.1002/mco2.491)
Supplement: Supplementary file 1 — Supporting information [file MCO2-5-e491-s001.docx]

**Maresin-1 ameliorates hypertensive vascular remodeling through its receptor LGR6**

**Supplemental Materials**


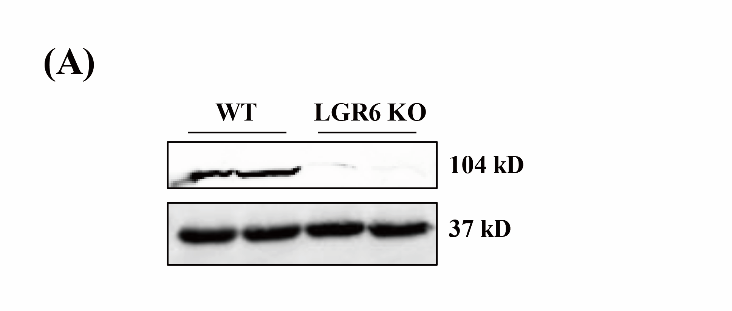


**Figure S1.** (A) Immunoblotting analysis of LGR6 in the aortas to verify LGR6 knockout efficiency (n=6).


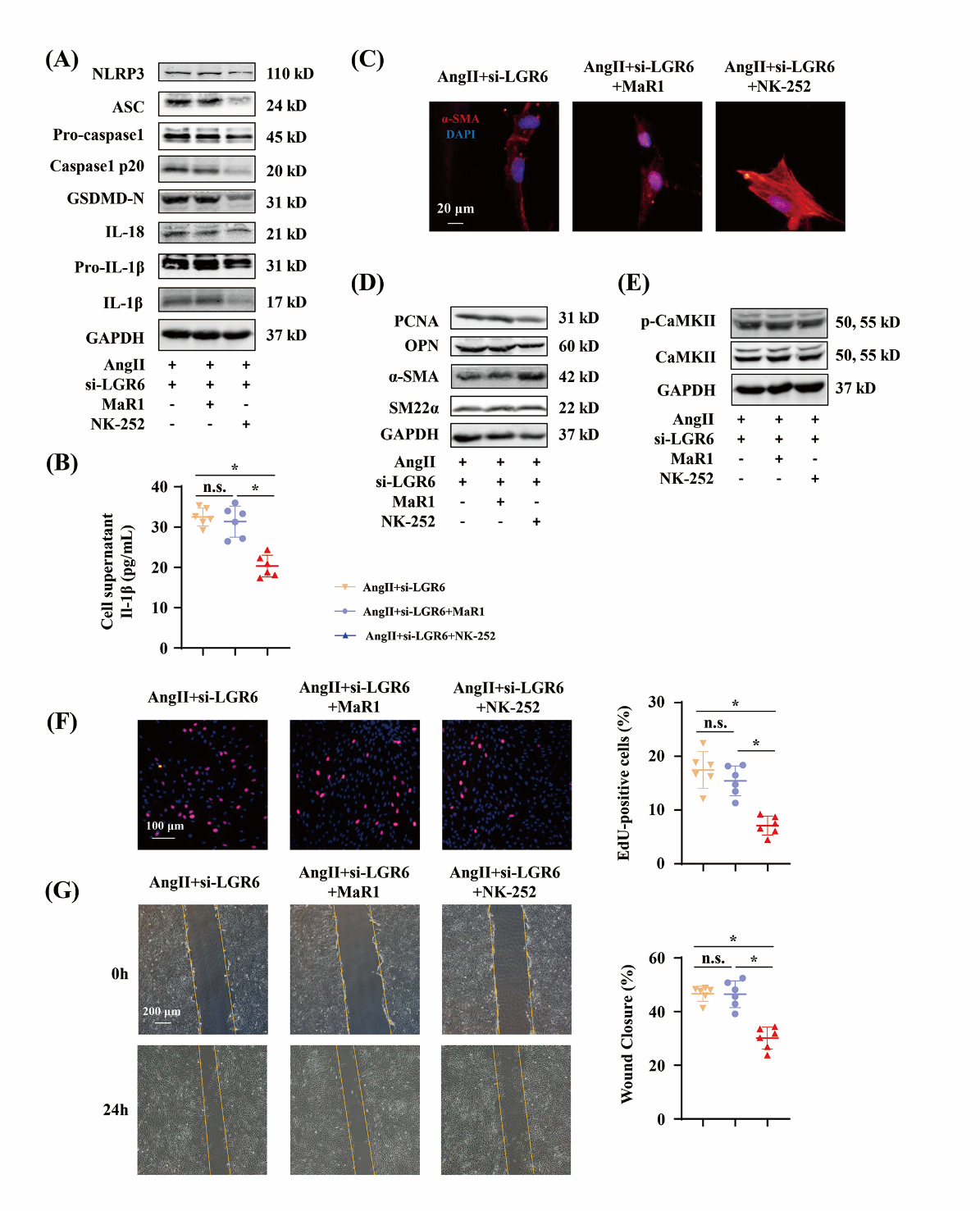


**Figure S2.** MaR1 serving as a Nrf2 activator requires the presence of its receptor LGR6 on VSMCs. (A) Immunoblotting analysis of NLRP3, ASC, pro-caspase1, caspase1 p20, GSDMD-N, IL-18, pro-IL-1β, and IL-1β in RASMCs transfected by siRNA in the presence or absence of MaR1(100 nM) or NK-252 (10 μM) for 24 h (n=6). (B) Quantitative analysis of Il-1β content in RASMC culture supernatant from the indicated groups (n=6). *P < 0.05, one-way ANOVA. (C) Representative images of α-SMA immunofluorescence staining of RASMCs from the indicated groups. (D) Immunoblotting analysis of PCNA, OPN, α-SMA, and SM22α in RASMCs from the indicated groups (n=6). (D) Immunoblotting analysis of p-CaMKII and CaMKII in RASMCs from the indicated groups (n=6). (F) Representative images of EdU staining of RASMCs from the indicated groups (n=6) and quantitative analysis. ∗p < 0.05, one-way ANOVA. (G) Representative images of wound healing of RASMCs from the indicated groups (n=6) and quantitative analysis. ∗p < 0.05, one-way ANOVA.
